# LINC00665 up-regulates SIN3A expression to modulate the progression of colorectal cancer via sponging miR-138-5p

**DOI:** 10.1186/s12935-021-02176-4

**Published:** 2022-01-31

**Authors:** Shoushan Nan, Shuangxia Zhang, Rong Jin, Juelei Wang

**Affiliations:** 1 Department of Gastroenterology, Tianjin Fifth Center Hospital, No. 41 Zhejiang Road, Binhai New District, Tianjin, 300450 China; 2 Department of Gastroenterology, Tianjin First Center Hospital, Tianjin, 300384 China; 3 Department of Gynaecology and Obstetrics, Tianjin Fifth Center Hospital, Tianjin, 300450 China

**Keywords:** LINC00665, miR-138-5p, SIN3A, Colorectal cancer

## Abstract

**Background:**

Colorectal cancer (CRC) is a malignant tumor affecting people worldwide. Long noncoding RNAs (lncRNAs) is a crucial factor modulating various cancer progression, including CRC. Long intergenic non-protein coding RNA 665 (LINC00665) has been proven as an oncogene in several cancers, but its function in CRC is still unclear.

**Methods:**

QRT-PCR was performed for RNA quantification. Functional assays were designed and carried to test cell phenotype while mechanism experiments were adopted for detecting the interaction of LINC00665, microRNA-138-5p (miR-138-5p) and SIN3 transcription regulator family member A (SIN3A). In vivo experiments were conducted to test LINC00665 function on modulating CRC tumor progression.

**Results:**

LINC00665 displayed high expression in CRC tissues and cells, and promoted tumor progression in vivo. MiR-138-5p displayed abnormally low expression in CRC, and was verified to be sponged by LINC00665. Furthermore, SIN3A, as the downstream mRNA of miR-138-5p, exerted promoting impacts on CRC cells. Rescue experiments certified that overexpressed SIN3A or silenced miR-138-5p could offset the repressed function of LINC00665 knockdown on CRC progression.

**Conclusions:**

LINC00665 could sponge miR-138-5p to up-regulate SIN3A expression, thus accelerating CRC progression.

**Graphic abstract:**

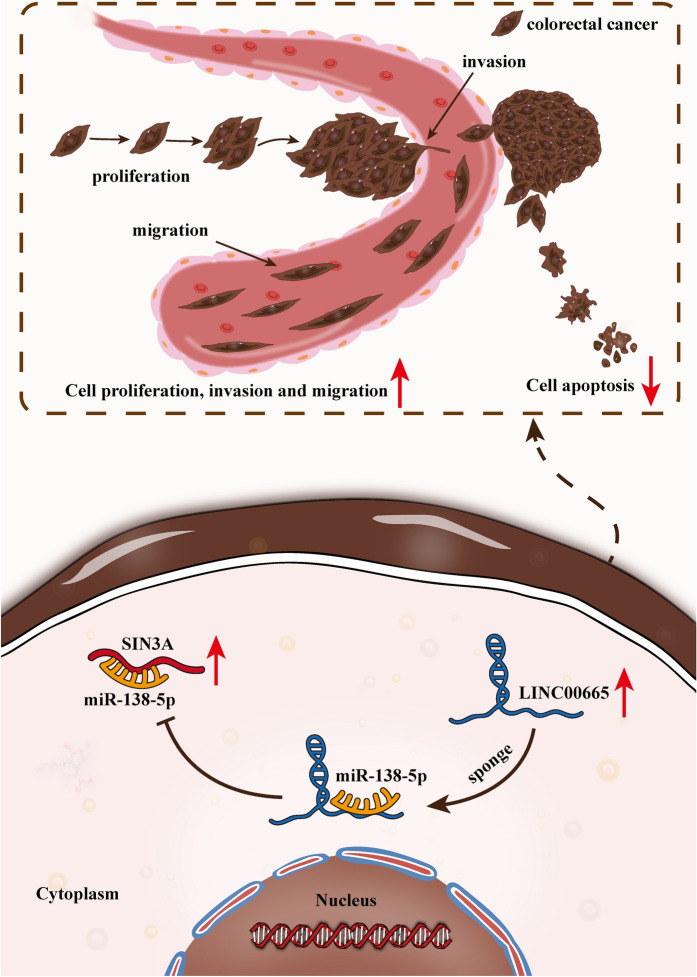

**Supplementary Information:**

The online version contains supplementary material available at 10.1186/s12935-021-02176-4.

## Background

Colorectal cancer (CRC) is a malignant tumor seriously threatening people’s health and quality of life [1]. Due to its high incidence, CRC is identified as the third most commonly diagnosed cancer worldwide [2]. The mortality rate of CRC is also high, as number of deaths caused by CRC ranks the fourth among all cancers [1]. In recent years, early screening methods and treatment strategies have been constantly developed, thereby improving the overall survival rate of CRC patients to a certain extent [3]. However, since the recurrence and metastasis frequently occur in most advanced patients, current treatment methods are less effective for those patients, and the survival rate and prognosis are not satisfactory [4, 5]. Thus, an in-depth investigation into the molecular mechanism of CRC will contribute to development of novel therapeutic targets, thus ultimately enhancing CRC patient survival rate.

Long noncoding RNAs (lncRNAs), possessing more than 200 nucleotides in length, are identified as RNA transcripts without the capability of coding proteins [6]. Recently, the crucial participation of lncRNAs in cellular behaviors including cell proliferation and apoptosis has been raveled out in mounting published studies [7, 8]. Importantly, the regulatory functions of lncRNAs in different cancers have also been increasingly investigated [9, 10]. LncRNA dysregulation has been confirmed as a critical factor in triggering human malignancies, and exerts promoting or inhibitory impacts on various cancers. For example, lncRNA AK021443 was found to be up-regulated in hepatocellular carcinoma cells and it could accelerate cell growth through regulating epithelial–mesenchymal transition [11]. GPR65-1 displayed abnormally high expression in gastric cancer cells and facilitated cell proliferation via PTEN-AKT-slug signaling pathway [12]. Moreover, Linc00662 was identified as a regulator in cell invasion and contributed to cancer stem cell-like phenotypes in lung cancer cells [13]. Additionally, long intergenic non-protein coding RNA 665 (LINC00665) has been proven to act as an oncogene in several cancers. For example, LINC00665 was reported to accelerate gastric cancer tumorigenesis by modulating miR-149-3p/RNF2 axis [14]. Also, LINC00665 was proven to display high expression in hepatocellular carcinoma cells and tissues, and could facilitate cell viability and autophagy while suppressing cell apoptosis via miR-186-5p/MAP4K3 axis in hepatocellular carcinoma [15]. Moreover, LINC00665 was confirmed to induce acquired resistance to gefitinib via regulating EZH2/PI3K/AKT pathway in non-small-cell lung cancer [16]. Nevertheless, the specific role and molecular mechanism of LINC00665 in CRC is unclear.

Herein, the foremost purpose of our research was to investigate the specific function and mechanism of LINC00665 in CRC, thus offering novel perspectives for exploration of CRC therapeutic targets.

## Methods

### Tissue samples

The tissue samples were obtained from Tianjin Fifth Center Hospital, with the ethical approval from Tianjin Fifth Center Hospital. The informed consent was acquired from participants prior to tissue collection. 60 pairs of CRC samples and adjacent non-tumor samples were excised from patients, and were immediately preserved in liquid nitrogen at − 80 °C.

### Cell culture

CRC cell lines (LOVO, HT-29, HCT116 and SW480) and human normal colorectal mucosa cell line (FHC) were bought from American Type Culture Collection (ATCC; Manassas, VA, USA). FHC was cultured in DMEM/F12 medium with 10% fetal bovine serum (FBS; Gibco, Grand Island, NY, USA) as the supplement. LOVO was cultivated in F-12 K medium with 10% FBS (Gibco). McCoy’s 5a medium supplemented with 10% FBS was utilized to culture HT-29 and HCT116 cells (Gibco). Leibovitz’s L-15 medium supplemented with 10% FBS was applied for the cultivation of SW480 cells. All cells were cultured in humidified environment with 5% CO_2_ at 37 °C.

### Cell transfection

The specific short hairpin RNA (shRNA) targeting LINC00665 and SIN3A, and negative control sh-NC were synthesized for down-regulating LINC00665 and SIN3A. Overexpressing SIN3A was achieved by utilizing pcDNA3.1-SIN3A. In addition, miR-138-5p mimics were applied for overexpressing miR-138-5p, and miR-138-5p inhibitor, the chemically designed, single-stranded RNA molecules, was utilized to eliminate miR-138-5p activity. All of the plasmids were procured from GenePharma (Shanghai, China). HCT116 and SW480 cells (1 × 10^6^) were transfected with the indicated plasmids respectively by utilization of Lipofectamine 3000 (Invitrogen) in accordance with the protocols of suppliers at a final concentration of 7 nM for 48 h. Each transfection experiment was independently repeated in triplicate. Sequences of transfected plasmids were listed in Additional file 4: Table S1.

### Quantitative real-time polymerase chain reaction (qRT-PCR) assay

On the basis the protocols of Trizol reagent (Invitrogen), the total RNA was extracted from HCT116 and SW480 cells (3 × 10^7^). In order to detect gene expressions, cDNA synthesis was accomplished by the utilization of PrimeScript™ RT reagent kit (Takara, Shiga, Japan). Later, qRT-PCR was conducted by means of SYBR Premix Ex Taq II (Takara). Finally, 2^−ΔΔCt^ method was applied for calculating the expression of detected genes. GAPDH or U6 served as control. The primers used for detecting LINC00665, miR-138-5p or SIN3A were listed in Additional file 5: Table S2. Raw data for qRT-PCR was provided in Additional file 6: Table S3.

### 5-Ethynyl-2′-deoxyuridine (EdU) assay

In this assay, we applied EdU incorporation assay kit (Ribobio, Guangzhou, China) for detecting cell proliferation in line with the manufacturer’s protocols. The transfected HCT116 and SW480 cells (1 × 10^5^) were collected and treated with 50 µL of EdU for 2 h at 37 °C. Then 4% paraformaldehyde was utilized to fix the cells, and cells were permeated by 0.5% Troxin X-100. After that, HCT116 and SW480 cells were cultivated with 1×Apollo®488 fluorescent dry reaction solution at 37 °C for half an hour. Then, DAPI was utilized to stain cell nucleus. Finally, we observed the colonies by a fluorescence microscope (Leica, Wetzlar, Germany).

### Transwell assay

The transfected HCT116 and SW480 cells (5 × 10^4^) were gathered and then re-suspended in serum-free medium and put into the upper chamber without Matrigel for detecting migration capability. For transwell invasion assay, the Matrigel was pre-coated in the upper chamber. Later, the lower chamber was added with 10% FBS. Twenty-four hours later, methanol was used to fix the migrated or invaded cells and crystal violet was applied to dye. Finally, the migrated or invaded cells were observed under microscope (Olympus Corp, Tokyo, Japan).

### Flow cytometry analysis

In accordance with the protocols of supplier, cell apoptotic capability was evaluated via the utilization of the Annexin V-FITC apoptosis kit (BD Biosciences, USA). Simply put, the transfected 4 × 10^6^ cells were collected and then rinsed by PBS for two times, followed by 10-min staining with Annexin V-FITC (BD Biosciences) in dark room. Flow cytometry (BD Biosciences, FACS Calibur) was applied for observation.

### JC-1 assay

For the sake of assessing the change in mitochondrial transmembrane potential (ΔΨm), HCT116 and SW480 cells in 96-well plates (2.5 × 10^5^/well) were cultivated for the whole night. After that, cells were centrifuged at room temperature for five minutes. Following mixing with 30-min JC-1 dye, cells were observed under fluorescence microscopy.

### Subcellular fractionation assay

PARIS™ Kit (Ambion, Austin, TX) was applied to determine the distribution of RNAs in nucleus and cytoplasm. HCT116 and SW480 cells (1 × 10^7^) were gathered and suspended again in the cell fraction buffer. Cells were then incubated on ice for ten minutes and centrifuged. Subsequent to removal of the upper solution, the nuclear pellet was kept for acquisition of RNA extracts by the utilization of a cell disruption buffer.

### Fluorescent in situ hybridization (FISH)

FISH assay was performed following the experiment protocols in previous report [17]. PBS was used to wash HCT116 and SW480 cells (5 × 10^4^) grown on the slides, followed by 30-min fixation with 4% paraformaldehyde. Then, the slides were treated with DNase to digest potentially contaminating DNA at 37 °C. Subsequent to incubation in prewarmed hybridization buffer at 55 °C for 2 h, the LINC00665 FISH probe or miR-138-5p FISH probe tagged by Cy3 (Ribobio) was added onto the culture dishes for hybridization at 65 °C overnight. After fostering with RNase A and blocking solution, the culture dishes were washed and treated with DAPI solution for staining. Finally, the stained cells were analyzed by fluorescent microscope. Sequences of LINC00665 biotin probe and miR-138-5p biotin probe were listed in Additional file 7: Table S4.

### RNA immunoprecipitation (RIP) assay

Magna RIP™ RNA-Binding Protein Immunoprecipitation Kit (EMD Millipore, MA, USA) was applied for conducting this assay. 6 × 10^7^ transfected cells were gathered and lysed with RIP lysis buffer. Then, RIP buffer treated cell lysates and magnetic beads conjugated with anti-Ago2 antibody (Cell Signaling Technology, USA, 1:50) or control anti-IgG (Cell Signaling Technology, 1:200) were utilized for incubation at 4 °C overnight. Following, immunoprecipitated RNA-protein complexes were cultivated with proteinase K at 55 °C for 30-min digestion. Then, co-precipitated RNA was eluted and purified by utilizing TRIzol™ Plus RNA Purification Kit (12183555, ThermoFisher, CA, USA) for qRT-PCR analysis.

### RNA pull down assay

The biotinylated DNA probe complementary to miR-138-5p wild type (miR-138-5p-Wt) or mutant (miR-138-5p-Mut) was devised and composed through Ribobio. HCT116 and SW480 cells were transfected with miR-138-5p-Wt or miR-138-5p-Mut. Biotinylated empty plasmids were taken as a control. The lysate obtained from 2 × 10^7^ cells was cultivated overnight with probe-coated beads (biotinylated probe and streptavidin magnetic beads) (Sigma-Aldrich). Then, they were washed by washing/binding buffer. Finally, the RNA complexes which combined with the beads were analyzed by qRT-PCR. Sequences involved in this assay were all listed in Additional file 8: Table S5.

### Luciferase reporter assay

1 × 10^4^ cells were involved in Luciferase reporter assay. The full length sequence of LINC00665 or SIN3A-3′UTR with wide-type or mutated miR-138-5p binding sites was obtained via chemical synthesis, which was respectively cloned into the pmirGLO dual-luciferase vectors later. Then, Lipofectamine 2000 (Invitrogen) was applied for co-transfection. 100 ng constructs were co-transfected with 7 nM NC mimics or miR-138-5p mimics into HCT116 and SW480 cells for 48 h. In the end, the relative luciferase activities were examined utilizing dual-luciferase reporter assay system (Promega, Madison, WI). Relevant sequences were all demonstrated in Additional file 9: Table S6.

### Western blot (WB) assay

The cells were lysed in cell lysis buffer. Proteins were extracted by sigma PROTTOT-1KT. Then, protein concentrations were measured using Bradford Protein Assay Kit. Later, SDS-PAGE was applied to isolate protein, and the protein was transferred to PVDF membrane, which was then co-cultured with specific primary antibodies, including anti-GAPDH (Cell Signaling Technology, 1:1000), and anti-SIN3A (Cell Signaling Technology, 1:1000). Secondary antibodies were later added to incubate the membranes for 1 h at 37 °C. In the end, the protein expression was measured by chemiluminescence detection system.

### Xenograft assay

Male BALB/c-nude mice of 4–5 weeks old were purchased from Institute of Zoology, Nanjing University. All animal experiment procedures were approved by Tianjin Fifth Center Hospital Hospital. The mice were randomly divided into two groups, CRC cells (1 × 10^7^) transfected with sh-LINC00665#1 and sh-NC were injected subcutaneously into the flank of nude mice. Tumor weight and volume were monitored every 4 days. Volume = (length × width^2^)/2. After 1 month, tumors were excised from all sacrificed mice.

### Statistical analysis

Each experiment in this study was independently conducted at least for three times. Statistics was analyzed by the utilization of GraphPad Prism 5.0 software. Data was represented as mean ± standard deviation (SD). The significance of the difference was evaluated via Student’s t-test, one-way analysis of variance (ANOVA) or two-way ANOVA. P < 0.05 was considered statistically significant in differences.

## Results

### Highly expressed LINC00665 facilitated cell proliferation, migration and invasion but impeded cell apoptosis in CRC

**Fig. 1:**
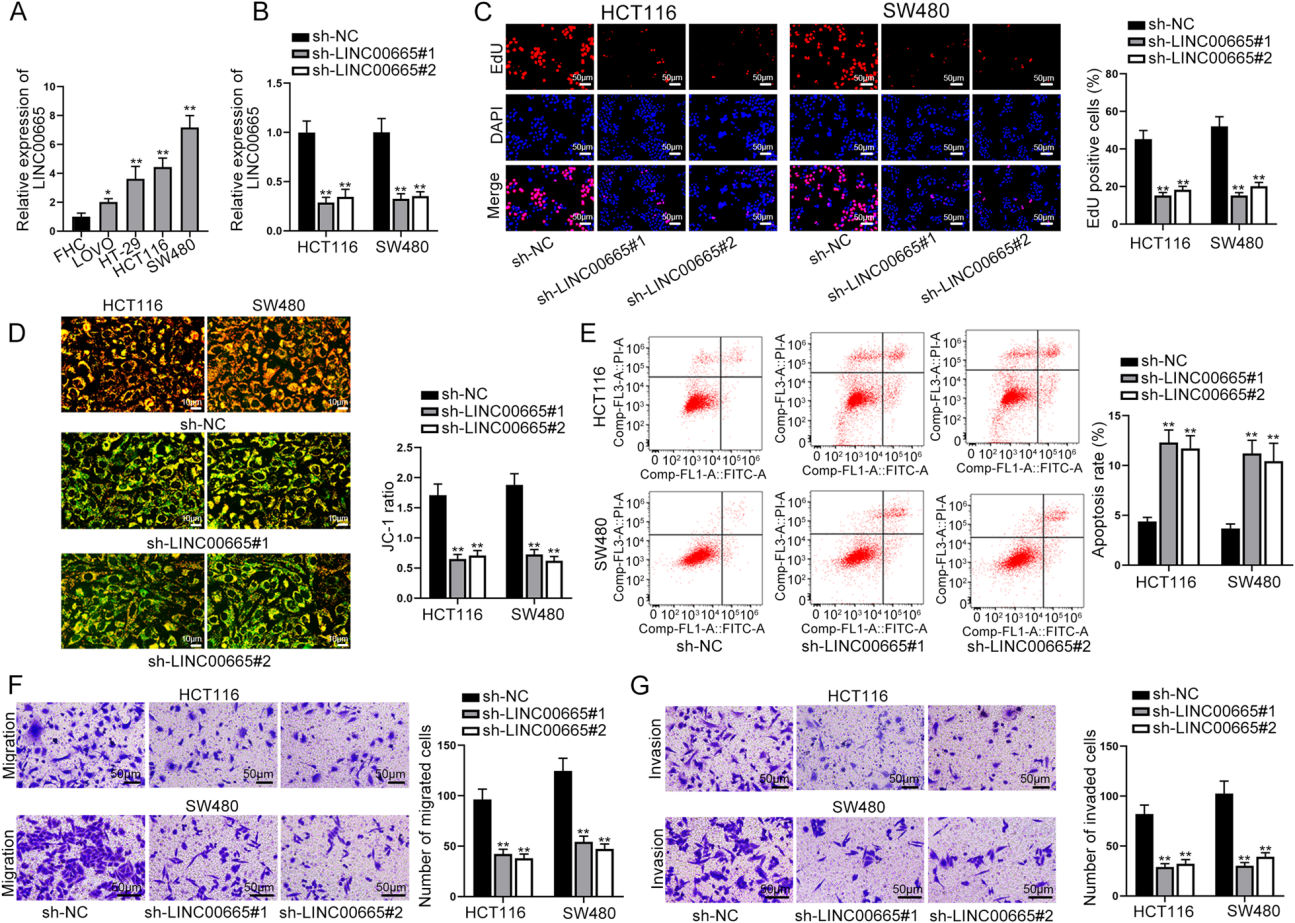
LINC00665 was found to exhibit high expression in CRC cells and modulated CRC cell growth, migration and invasion. **A** Quantification of LINC00665 expression in different cell lines was conducted via qRT-PCR. **B** The interference efficacy of sh-LINC00665 in cells was determined via qRT-PCR. **C** EdU experiments were conducted to examine cell proliferation after LINC00665 silence (×100 magnification). **D** JC-1 detected cell apoptosis under conditions of sh-LINC00665 transfection (×200 magnification). **E** Flow cytometry was performed to test cell apoptosis under the condition of sh-LINC00665 transfection. **F**, **G** Cell migration and invasion was testified via transwell assays in response to inhibition of LINC00665 (×100 magnification). *P < 0.05, **P < 0.01

First, we utilized qRT-PCR for determining LINC00665 expression in tumor and non-tumor samples (Additional file 1: Fig. S1A). LINC00665 expression was additionally analyzed in different cell lines. Specifically, we utilized qRT-PCR analysis to quantify LINC00665 expression in CRC cell lines (LOVO, HT-29, HCT116 and SW480) and human normal colorectal mucosal cell line (FHC). We discovered a remarkable up-regulation of LINC00665 in CRC cell lines, particularly in HCT116 and SW480 cells (Fig. 1A). After transfecting shRNAs targeting LINC00665 into cells, qRT-PCR confirmed the successful depletion of LINC00665. LINC00665 expression could be evidently reduced after transfection (Fig. 1B). Afterwards, we implemented the functional experiments to investigate the role of LINC00665 knockdown in modulating cell behaviors. Through EdU experiments, we discovered that EdU positive cells declined after knockdown of LINC00665, indicating LINC00665 depletion could lead to lessened cell proliferation (Fig. 1 C). The results of JC-1 and flow cytometry displayed that JC-1 ratio reduced and apoptosis rate was elevated when LINC00665 was silenced, which demonstrated that LINC00665 deficiency could accelerate cell apoptosis (Fig. 1D, E). In addition, it was manifested through transwell assays that cell migration and invasion could be hampered by LINC00665 down-regulation (Fig. 1F, G). Taken together, in vitro experiments evidenced that excessive high level of LINC00665 in CRC cells resulted in aggravated cell growth, migration and invasion. For further investigation into LINC00665 function in CRC progression, in vivo experiments were conducted by injecting mice with sh-LINC00665#1 or sh-NC transfected CRC cells. The result displayed that LINC00665 knockdown inhibited the progression of CRC in nude mice significantly (Additional file 1: Fig. S1B, C).

### MiR-138-5p was down-regulated in CRC and sponged by LINC00665

**Fig. 2:**
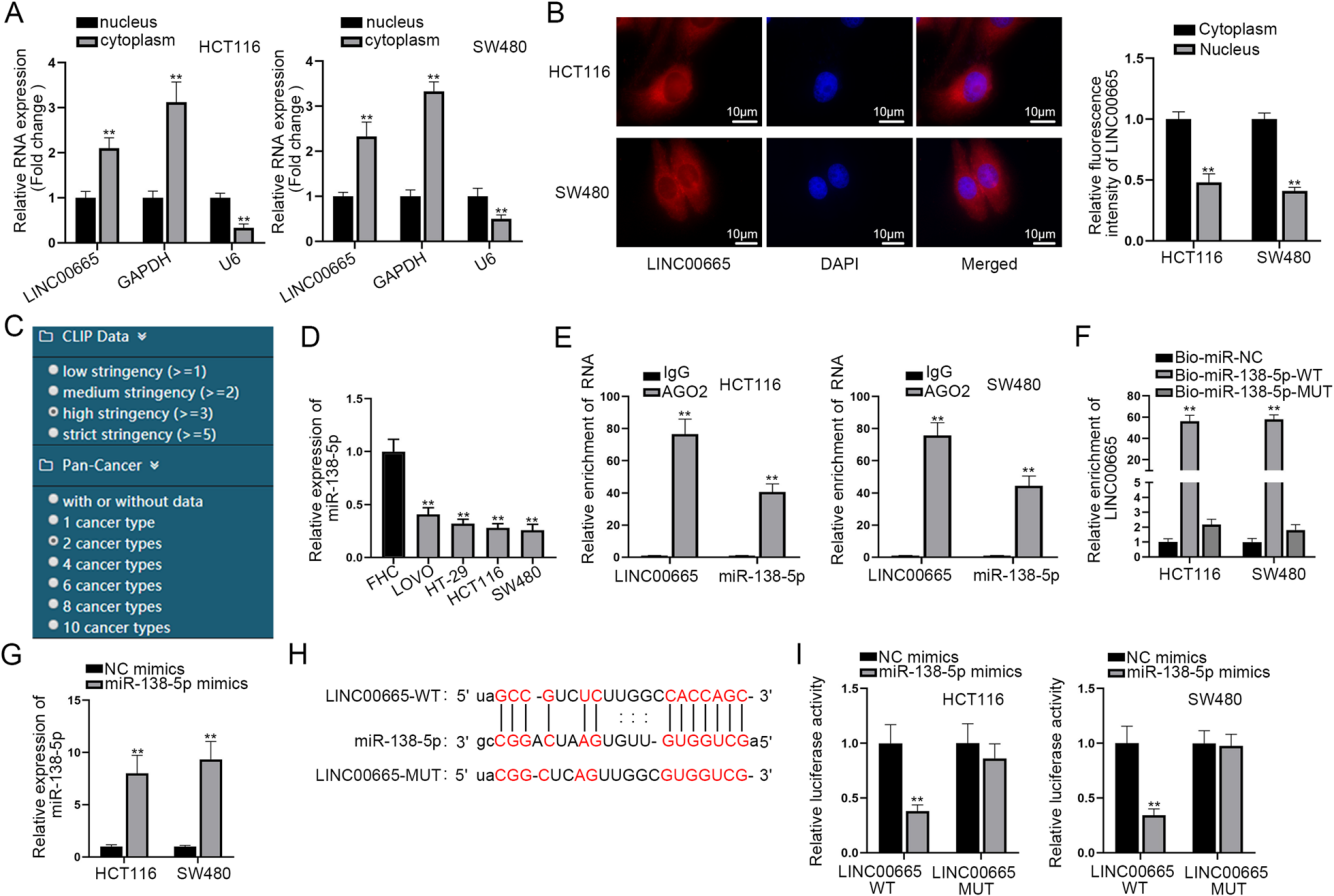
MiR-138-5p was sponged by LINC00665 in CRC cells. **A** Subcellular fractionation was conducted to test the distribution of LINC00665 in HCT116 and SW480 cells. **B** FISH assay was performed and co-localization profile intensity analysis was performed (×1000 magnification). **C** The screening conditions of miRNAs on ENCORI were demonstrated. **D **qRT-PCR was applied for the calculation of miR-138-5p expression in CRC cells. **E** RIP assay was carried out for verifying the relationship of miR-138-5p and LINC00665. **F** RNA pull down experiment was conducted to prove the combination of LINC00665 and miR-138-5p. **G** The overexpression efficacy of miR-138-5p mimics was determined through qRT-PCR. **H** The binding site of miR-138-5p and LINC00665 was demonstrated. **I** Luciferase reporter experiment verified the interaction of miR-138-5p and LINC00665. **P < 0.01

Then, for investigation of the regulatory mechanism of LINC00665 in CRC, we firstly detected LINC00665 distribution in HCT116 and SW480 cells through subcellular fractionation assay and FISH assay. The results manifested LINC00665 mainly distributed in cell cytoplast (Fig. 2A, B), implying that LINC00665 may exert its function post-transcriptionally. Competing endogenous RNA (ceRNA) network is a regulatory mechanism at post-transcriptional level and is discovered in assorted cancers through pieces of research [18]. LncRNA could sponge microRNAs (miRNAs) to regulate messenger RNA (mRNA) expression by serving as ceRNA [19]. Thus, we searched on ENCORI (http://starbase.sysu.edu.cn/) for prediction of potential downstream miRNA of LINC00665. Under the specific conditions (CLIP-Data ≥ 3, pan-Cancer ≥ 2), we found twenty miRNAs that possibly bound with LINC00665 (Fig. 2C). It was leant from the RNA pull down outcomes that miR-138-5p, miR-744-5p and miR-3140-3p could be significantly pulled down by LINC00665 biotin probe (Additional file 2: Fig. S2A). Then qRT-PCR analysis was done for detecting the expressions of miR-138-5p, miR-744-5p and miR-3140-3p in CRC cell lines for further screening. The results evidenced that the expression of miR-744-5p and miR-3140-3p in CRC cell lines was not significantly different from that in human normal colorectal mucosal cell line (FHC) (Additional file 2: Fig. S2B). However, miR-138-5p expression in CRC cell lines was obviously lower in comparison with that in FHC (Fig. 2D). MiR-138-5p expression in clinical samples was subsequently investigated via qRT-PCR analysis, and consistently, miR-138-5p displayed lower expression in CRC tissues than in non-tumor tissues (Additional file 2: Fig. S2C). Then, FISH assay was implemented again to co-locate miR-138-5p and LINC00665. The result implied that both miR-138-5p and LINC00665 mostly existed in cytoplasm (Additional file 2: Fig. S2D). Subsequently, we implemented RIP assay to detect the binding correlation between miR-138-5p and LINC00665. It was reflected in the outcomes that both miR-138-5p and LINC00665 were precipitated in Ago2 group (Fig. 2E). Based on outcomes of RNA pull down assay, LINC00665 could be precipitated in the pull-down of biotinylated miR-138-5p-WT, which reflected the binding correlation between miR-138-5p and LINC00665 (Fig. 2F). Then it was suggested in qRT-PCR analysis that the transfection of miR-138-5p mimics resulted in a remarkable increment in miR-138-5p expression (Fig. 2G). After obtaining the binding sites of LINC00665 to miR-138-5p, we carried out luciferase reporter experiments for evaluating the combination of miR-138-5p and LINC00665 (Fig. 2H, I). The result further verified that miR-138-5p overexpression could reduce the luciferase activity of LINC00665-WT, while that of LINC00665-Mut was almost unchanged under the same condition. These experimental results demonstrated that LINC00665 could directly bind to miR-138-5p. Taken together, we confirmed that lowly expressed miR-138-5p in CRC could be sponged by LINC00665.

### LINC00665 sequestered miR-138-5p to promote CRC cell proliferation, migration and invasion, but repressed cell apoptosis

**Fig. 3:**
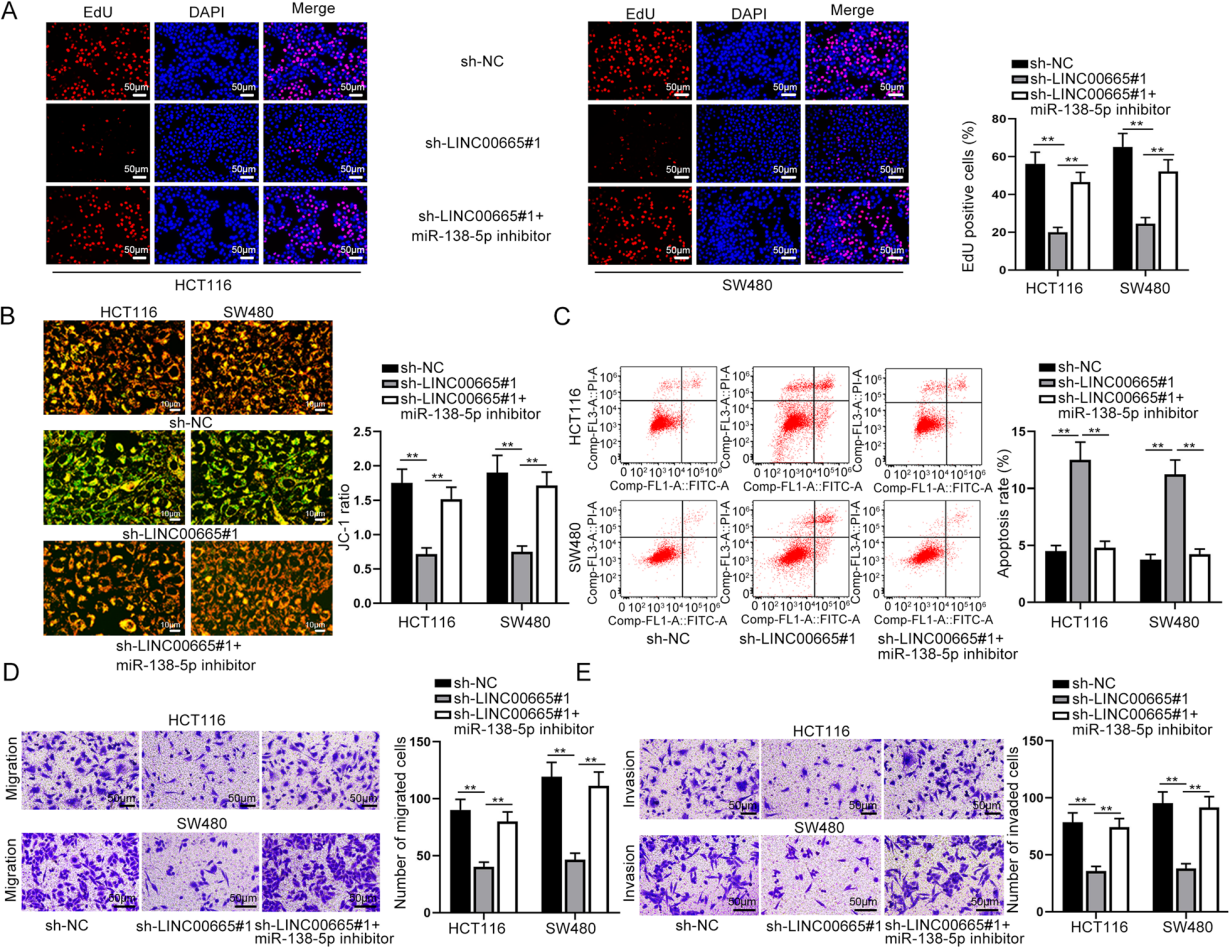
LINC00665 promoted CRC cell growth, migration and invasion via sponging miR-138-5p. **A** The proliferative capacity of CRC cells transfected with different plasmids (sh-NC, sh-LINC00665#1, and sh-LINC00665#1 + miR-138-5p inhibitor) was investigated via EdU (×100 magnification). **B**, **C** JC-1 (×200 magnification) and flow cytometry assays were carried out to measure apoptosis under different transfection conditions. **D**, **E** Migrated or invaded cells was observed and analyzed through transwell experiments (×100 magnification). **P < 0.01

For the evaluation of the interaction between LINC00665 and miR-138-5p in CRC cells, we conducted the rescue functional experiments. First of all, it was learnt from the experimental results of EdU assay that LINC00665 depletion notably weakened cell proliferative ability, which was then reversed in response to miR-138-5p inhibitor co-transfection (Fig. 3A). Then it was demonstrated through JC-1 and flow cytometry assays that cell apoptosis could be accelerated when LINC00665 was knocked down, and the trend was offset by miR-138-5p depletion (Fig. 3B, C). In addition, we discovered in transwell experiments that the repressed cell migration and invasion capabilities resulting from LINC00665 silence were reversed in response to miR-138-5p depletion (Fig. 3D, E). Taken together, LINC00665 could expedite CRC cell growth while mitigating cell apoptosis via functioning as miR-138-5p sponge.

### SIN3A was the target of miR-138-5p in CRC

**Fig. 4:**
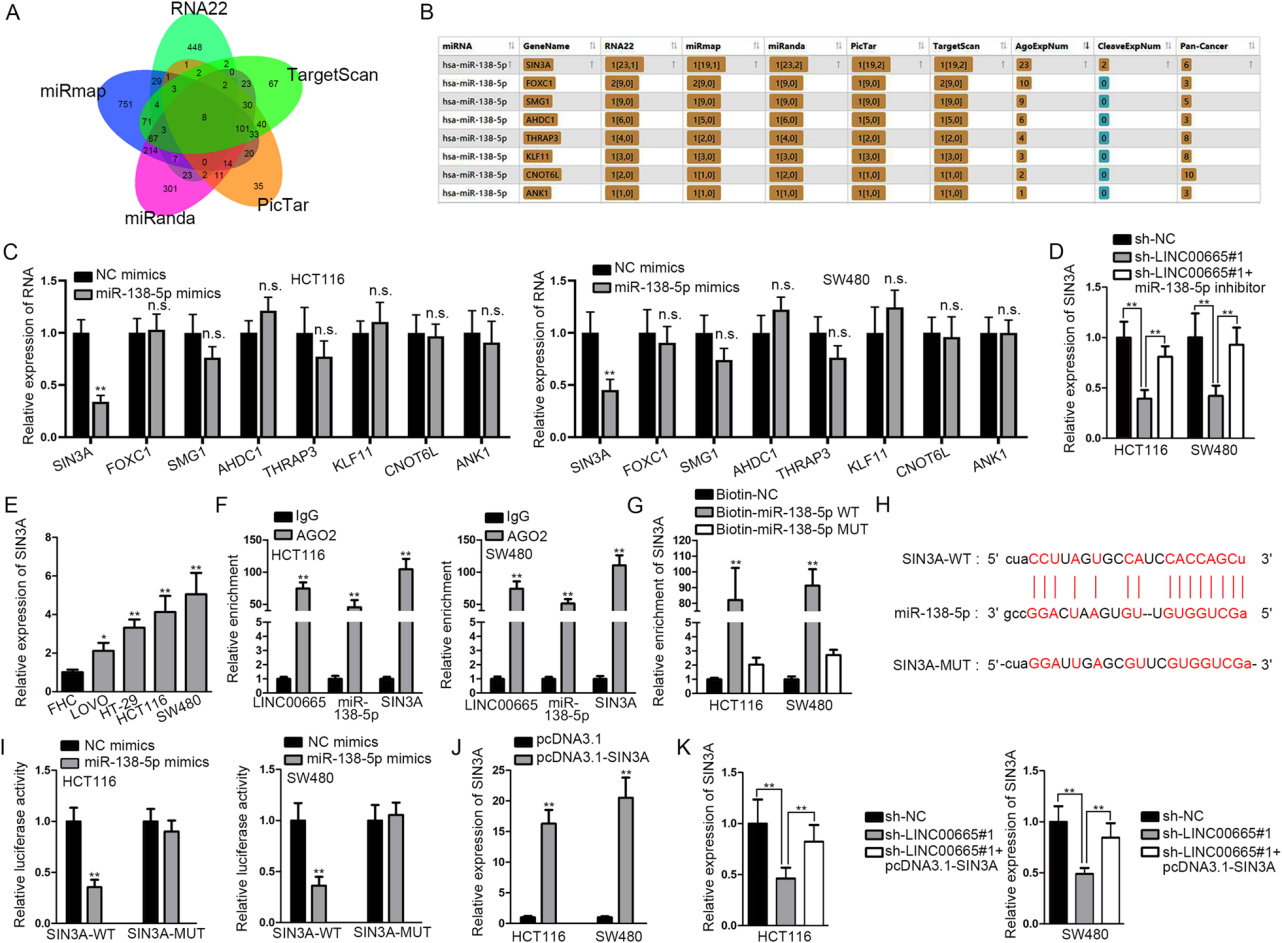
SIN3A was the downstream mRNA of miR-138-5p in CRC cells.   **A**, **B** 8 possible mRNAs were predicted through RNA22, miRmap, miRanda, PicTar and TargetScan databases. **C** qRT-PCR detected expression of the 8 mRNAs in cells after miR-138-5p mimics transfection. **D** The expression of SIN3A was quantified in cells with transfection of indicated plasmids via qRT-PCR. **E** SIN3A expression in CRC cell lines and FHC was measured via qRT-PCR. **F**, **G** RIP and RNA pull down assay were done for evaluating the relationship of LINC00665, miR-138-5p and SIN3A. **H** The binding site of SIN3A and miR-138-5p was obtained on ENCORI. **I** Interaction of SIN3A and miR-138-5p was reflected through luciferase reporter experiment. **J** SIN3A overexpression efficacy of was tested by qRT-PCR. **K** SIN3A expression was quantified by applying qRT-PCR in cells with indicated transfection. *P < 0.05, **P < 0.01, *n.s* no significance

For further exploring the regulatory mechanism in CRC, we searched for the possible downstream mRNA of miR-138-5p. After utilizing ENCORI, we found 8 mRNAs which could bind to miR-138-5p under the prediction of RNA22, miRmap, miRanda, PicTar and TargetScan databases (Fig. 4A, B). Then qRT-PCR was done for measuring the expression of 8 mRNAs (SIN3A, FOXC1, SMG1, AHDC1, THRAP3, KLF11, CNOT6L and ANK1) in HCT116 and SW480 cells with miR-138-5p mimics transfection (Fig. 4C). According to qRT-PCR analysis, SIN3A was down-regulated in cells after overexpressing miR-138-5p, while the expression levels of other mRNAs were hardly affected by miR-138-5p mimics. Thus, we chose SIN3A to perform following experiments. Then SIN3A expression was quantified in cells with sh-LINC00665 transfection or co-transfection of sh-LINC00665 and miR-138-5p, and according to the results, the decline in SIN3A expression as a result of LINC00665 depletion could be recovered on account of miR-138-5p inhibition (Fig. 4D). Afterwards, qRT-PCR analysis was done for the evaluation of SIN3A expression in CRC cells and FHC, and the results indicated that SIN3A expression was obviously higher in CRC cells in comparison with that in FHC cells (Fig. 4E). Additionally, SIN3A was up-regulated in CRC tissues relative to non-tumor tissues based on results of qRT-PCR and WB analysis (Additional file 3: Fig. S3A, B). RIP assay was conducted to evaluate the relationship among LINC00665, miR-138-5p and SIN3A and the results displayed that all of them were co-precipitated in RNA-induced silencing complex (RISC) (Fig. 4F). Then according to outcomes of RNA pull down assay, SIN3A was precipitated in biotinylated miR-138-5p-WT, indicating SIN3A bound with miR-138-5p (Fig. 4G). Additionally, we obtained the binding sites of miR-138-5p and SIN3A on ENCORI (Fig. 4H). The luciferase reporter assay was conducted to further verify the interaction between them. The results indicated that the luciferase activity of SIN3A-WT could be hampered as a result of miR-138-5p up-regulation, but the activity of SIN3A-Mut was hardly affected under the same condition (Fig. 4I). In addition, after determining the efficiency of pcDNA3.1-SIN3A (Fig. 4J), it was observed that reduction in SIN3A expression resulting from LINC00665 depletion was recovered by overexpressed SIN3A (Fig. 4K). A series of loss-of-function assays were done for verifying the influence of SIN3A depletion on CRC cells. After determining the SIN3A knockdown efficiency via qRT-PCR and WB assays (Additional file 3: Fig. S3C, D), it was reflected that SIN3A depletion hindered CRC cell proliferation, migration and invasion but promoted cell apoptosis (Additional file 3: Fig. S3E–I). Overall, SIN3A was the downstream mRNA of miR-138-5p, positively modulating CRC cell proliferation, migration and invasion, while negatively regulating CRC cell apoptosis.

### LINC00665 accelerated the CRC progression by up-regulating SIN3A

**Fig. 5:**
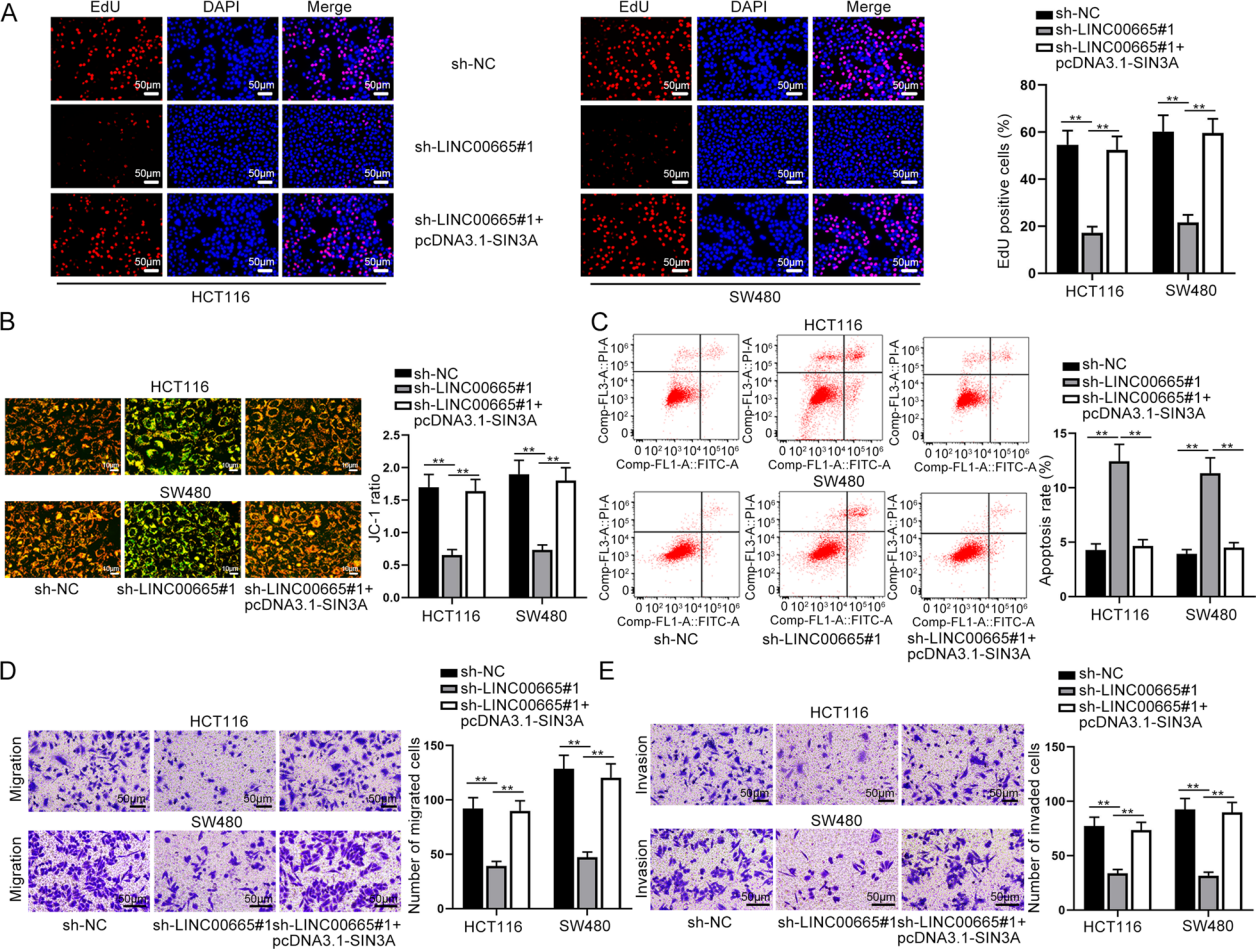
LINC00665 accelerated proliferation, migration and invasion of CRC cells and impeded cell apoptosis by up-regulating SIN3A. **A** EdU experiments were performed to estimate cell proliferation in response to transfection with indicated plasmids including sh-NC, sh-LINC00665#1, and sh-LINC00665#1 + pcDNA3.1-SIN3A (×100 magnification). **B**, **C** Cell apoptosis was evaluated by JC-1 (×200 magnification) and flow cytometry experiments under different transfection conditions. **D**, **E** Transwell assays were implemented to detect cell migration and invasion in different groups (×100 magnification). **P < 0.01

Finally, we probed into the impacts of LINC00665/SIN3A axis on biological behaviors of CRC cells through rescue assays. From EdU assays, it was reflected that LINC00665 knockdown notably weakened cell proliferative capacity, which was counteracted by SIN3A overexpression (Fig. 5A). Moreover, JC-1 and flow cytometry experiment indicated that the strengthened cell apoptotic ability was fully countervailed in response to overexpressed SIN3A (Fig. 5B, C). In the end, based on results of transwell assays, the inhibitory impacts of LINC00665 silence on cell migration and invasion could be neutralized as a result of SIN3A up-regulation (Fig. 5D, E). In a word, we proved that LINC00665 accelerated proliferation, migration and invasion of CRC cells but impeded CRC cell apoptosis by up-regulating SIN3A.

## Discussion

CRC is among the most common malignancies that incur serious health burden for people across the world. Recently, lncRNAs have been widely studied by scholars for their unique regulatory functions in modulating malignancies, including CRC. Specifically, TUG1 could expedite CRC cell metastasis and epithelial-mesenchymal transition via the regulation of KIAA1199/miR-600 [20]. HOTAIR was confirmed to regulate the progression and chemoresistance of CRC through miR-203a-3p-mediated Wnt/ß-catenin signaling pathway [21]. Additionally, SNHG8, up-regulated in colorectal cancer tissues and cells, was testified to exert its tumor promoting impacts on colorectal cancer through sponging miR-663 [22]. In our research, we investigated LINC00665 in CRC, which had been confirmed to exert the carcinogenic effect on gastric cancer [14], hepatocellular carcinoma [15] and non-small-cell lung cancer [16]. Aberrantly high expression of LINC00665 was discovered in tissues of these three cancers, and consistently, we discovered that LINC00665 was also overexpressed in CRC tissues. Moreover, LINC00665 expression was also measured in CRC cell lines via qRT-PCR, and LINC00665 was discovered to be up-regulated in CRC cell lines. So we suspected that LINC00665 also exerted carcinogenic effect on CRC. After that, we implemented functional experiments to scrutinize the function of LINC00665 in CRC and discovered that knockdown of LINC00665 could lead to restrained CRC cell proliferation, migration and invasion while contributing to strengthened cell apoptosis. Moreover, it was learnt from in vivo experiments that LINC00665 depletion could impede tumor growth. Thence, the oncogenic role of LINC00665 in CRC was raveled out, which was consistent with former findings that LINC00665 could promote the progression of CRC [23].

Furthermore, it was proved that LINC00665 was mainly accumulated in cytoplasm of CRC cells through subcellular fractionation and FISH assays. Thus, we predicted that LINC00665 may exert its function in CRC as a ceRNA. Over the past decades, lncRNAs have been reported to modulate mRNA expression through serving as sponge of certain miRNA [18]. The ceRNA network is a regulatory mechanism for the interaction between RNAs in the cytoplasm at post-transcriptional regulation level and is discovered in assorted cancers [19, 24]. For example, XIST was reported to facilitate cervical cancer progression through up-regulating Fus via competitively binding with miR-200a [25]. FLVCR1-AS1 could conduce to lung cancer cell growth through sponging miR-573 to regulate E2F3 expression [26]. Moreover, SNHG15 was confirmed to regulate YAP1-Hippo signaling pathway via sequestering miR-200a-3p in papillary thyroid carcinoma [27].

In our current research, after LINC00665 was proved as a cytoplasmic lncRNA in CRC cells, we utilized the bioinformatics tool for the prediction of the possible miRNA for LINC00665. Through ENCORI database, we discovered that twenty miRNAs could potentially bind to LINC00665. Through RNA pull down assay and qRT-PCR analysis, miR-138-5p was picked out. MiR-138-5p was low expressed in CRC cells and tissues, and it was demonstrated through qRT-PCR analysis and mechanism experiments including RIP, RNA pull down and luciferase reporter assays that miR-138-5p could be sponged by LINC00665 in CRC cells. Previous research confirmed that miR-138-5p frequently served as the tumor suppressor in some cancers. For example, miR-138-5p was ascertained to suppress autophagy in pancreatic cancer through targeting SIRT1 [28]. Additionally, miR-138-5p, down-regulated in lung adenocarcinoma tissues and cells, was ascertained to repress metastasis via targeting ZEB2 [29]. Also, miR-138-5p could restrain the progression of CRC by targeting PD-L1 [30]. Based on results of current function assays in a rescue manner, miR-138-5p inhibition could countervail the suppressive impacts of LINC00665 depletion on cell proliferation, migration and invasion, as well as the promoting influence of LINC00665 silence on cell apoptosis. Taken together, LINC00665 could sponge miR-138-5p to play its oncogenic role in CRC.

Next, bioinformatics tool was applied for the prediction of downstream mRNA of miR-138-5p and SIN3A was found. Based on qRT-PCR results, SIN3A displayed high expression in CRC cells and tissues. And through mechanism experiments, SIN3A was verified as the target gene of miR-138-5p. According to former studies, SIN3A could affect gene expression at epigenetic level, thereby affecting the progression of cancer cells [31]. Previous studies also offered possible explanation of mechanistic role of SIN3A. For example, Giovanni Gambi, et al. discovered SIN3A was a regulator of STAT3 and identified the STAT3/SIN3A axis as a possible target to deal with STAT3-addicted tumors [32]. SIN3A was suggested to affect breast cancer progression, and the correlation between SIN3A mRNA expression and the recurrence of ER-positive breast cancer was also raveled out [33]. In addition, Yang, et al. verified SIN3A/HDAC/LSD1 axis could regulate chemo-resistance in breast cancer [34]. MiR-210/SIN3A axis was also validated to facilitate glioma cell apoptosis [35]. SIN3A was discovered to be overexpressed in CRC cells and tissues. Additionally, in current loss-of-function assays, CRC cell proliferation, migration and invasion were overtly hampered in response to SIN3A silence, while CRC cell apoptosis was facilitated on account of the decline in SIN3A expression. Furthermore, rescue experiments indicated that the inhibitory impacts of LINC00665 silence on CRC cell growth, migration and invasion could be countervailed on account of SIN3A overexpression, and the strengthened cell apoptosis induced by LINC00665 depletion was also reversed by SIN3A up-regulation.

Taken together, our study revealed that LINC00665 could up-regulate SIN3A expression to accelerate the CRC progression via sponging miR-138-5p, which has not been discussed before. LINC00665 was validated to facilitate CRC cell growth and tumor progression via modulating miR-138-5p/SIN3A, which may offer strong evidence that LINC00665 might act as a potential therapeutic target in CRC treatment.

## Conclusions

Our study investigated the molecular mechanism of LINC00665 in CRC. We firstly revealed that LINC00665 could up-regulate SIN3A to facilitate CRC cell growth, migration and invasion via sponging miR-138-5p and the promoting impacts of LINC00665 on CRC progression was also confirmed, which might contribute to the discovery of potential novel therapeutic targets for CRC.

## Supplementary Information

**Additional file 1: Figure S1.** (A) The expression of LINC00665 in patient samples was detected by qRT-PCR. (B, C) In vivo experiment was carried out and the growth of tumor was monitored. Tumor volume and weight were also calculated. **P < 0.01.

**Additional file 2: Figure S2.** (A) RNA pull down assay was utilized to screen out the miRNAs which could bind with LINC00665. (B) The qRT-PCR analysis was conducted to detect the expressions of miR-744-5p and miR-3140-3p in different cell lines. (C) The expression of miR-138-5p in patient samples was detected by qRT-PCR. (D) FISH assays were conducted for determining the localization of miR-138-5p and LINC00665 in HCT116 and SW480 cells (×1000 magnification). **P < 0.01, n.s.: no significance.

**Additional file 3: Figure S3.** (A, B) The expression of SIN3A in patient samples was measured by qRT-PCR and WB. (C, D) qRT-PCR and WB were carried out to evaluate the knockdown efficiency of sh-SIN3A. (E) EdU experiments were performed to estimate cell proliferation in response to SIN3A depletion (×100 magnification). (F, G) Cell apoptosis was evaluated by JC-1 (×200 magnification) and flow cytometry experiments in different groups. (H, I) Transwell assays were implemented to detect cell migration and invasion in different groups (×100 magnification). **P < 0.01.

**Additional file 4: Table S1.** The sequences of transfection plasmids were listed.

**Additional file 5: Table S2.** The primer sequences used in qRT-PCR were listed.

**Additional file 6: Table S3.** The sequences of FISH probes targeted LINC00665 and miR-138-5p were listed.

**Additional file 7: Table S4.** Antibodies of RIP assay were listed.

**Additional file 8: Table S5.** The sequences used in RNA pull down assay were listed.

**Additional file 9: Table S6.** The sequences used in luciferase reporter assay were listed.

## Data Availability

Research data are not shared.

## Abbreviations

CRC: Colorectal Cancer
LINC00665: Long intergenic non-protein coding RNA 665
SIN3A: SIN3 transcription regulator family member A
lncRNAs: Long noncoding RNAs
mRNAs: Messenger RNAs
miRNAs: MicroRNAs
ceRNA: Competing endogenous RNA
ATCC: American Type Culture Collection
shRNA: Short hairpin RNA
NC: Negative control
qRT-PCR: Quantitative real-time polymerase chain reaction
EdU: 5-Ethynyl-2′-deoxyuridine
FISH: Fluorescence in situ hybridization
RIP: RNA immunoprecipitation
WT: Wild-type
Mut: Mutant
WB: Western blot
SD: Standard deviation
ANOVA: Analysis of Variance
