# Supplementary figures and images for: LINC00665 up-regulates SIN3A expression to modulate the progression of colorectal cancer via sponging miR-138-5p
Source: Cancer Cell Int. 2022 Jan 31;22:51. doi: 10.1186/s12935-021-02176-4 (PMC8802510; doi:10.1186/s12935-021-02176-4)

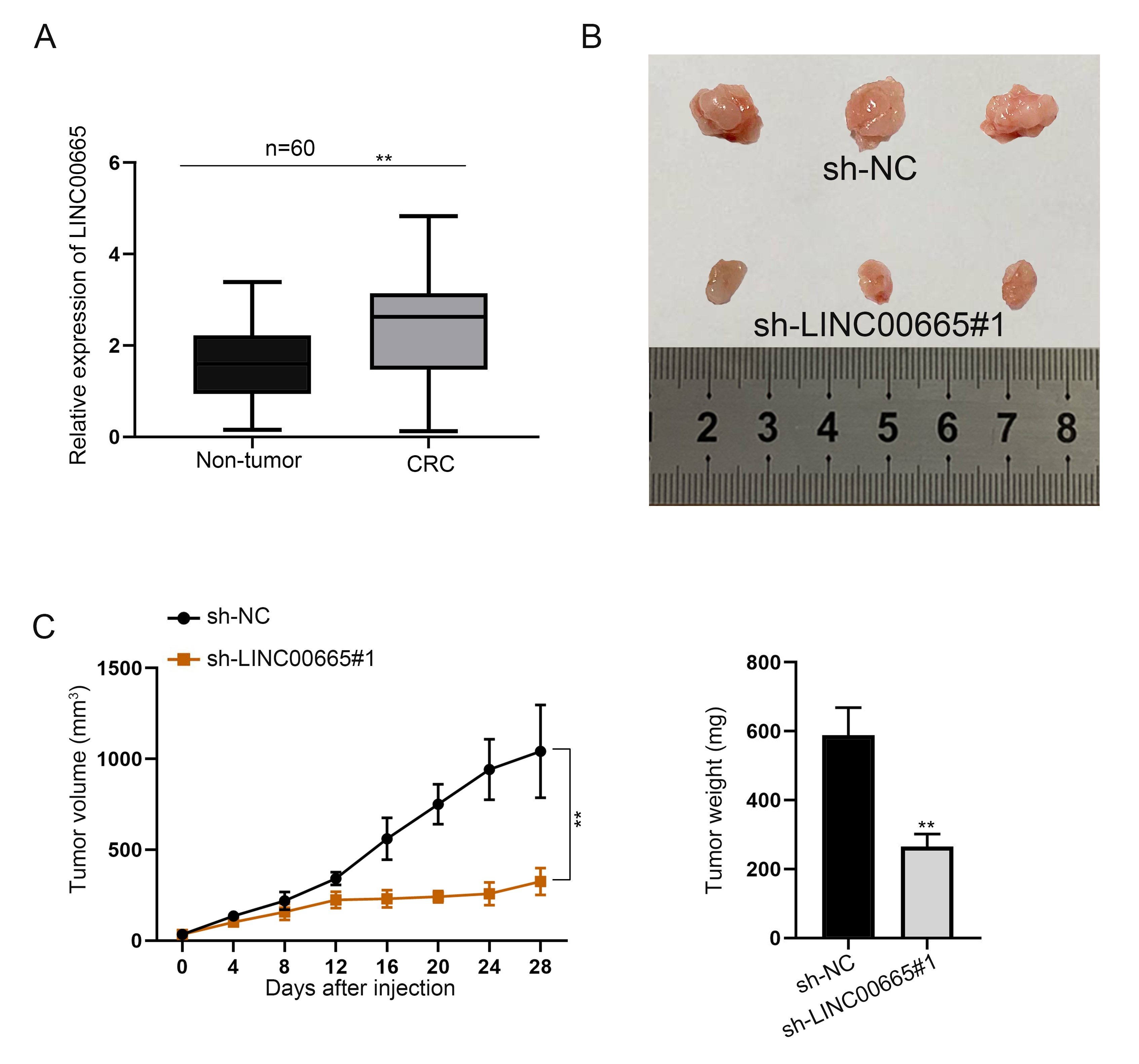

Supplement: Supplementary file 1 — Additional file 1: Figure S1. (A) The expression of LINC00665 in patient samples was detected by qRT-PCR. (B, C) In vivo experiment was carried out and the growth of tumor was monitored. Tumor volume and weight were also calculated. **P < 0.01. [file 12935_2021_2176_MOESM1_ESM.tif]

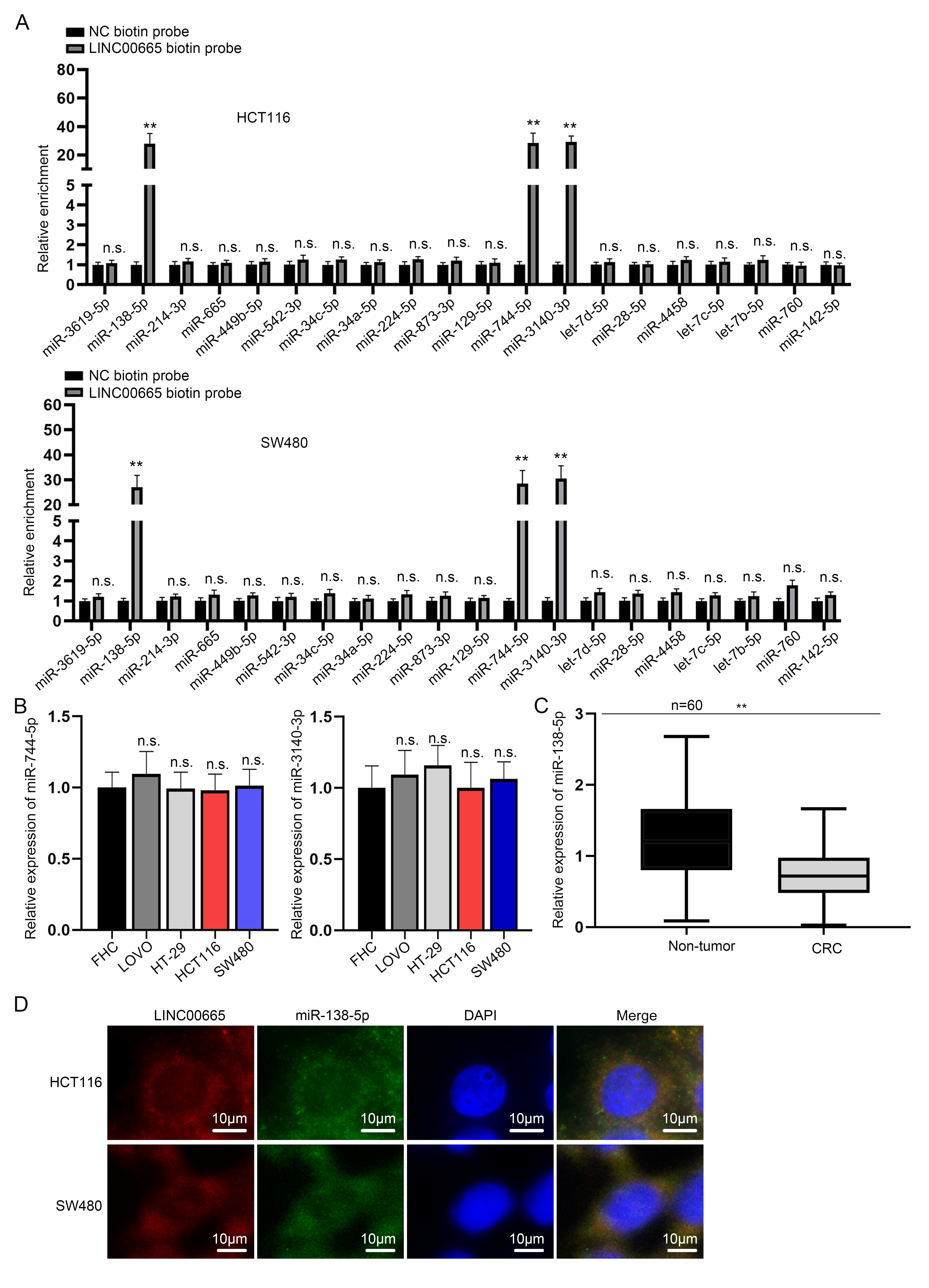

Supplement: Supplementary file 2 — Additional file 2: Figure S2. (A) RNA pull down assay was utilized to screen out the miRNAs which could bind with LINC00665. (B) The qRT-PCR analysis was conducted to detect the expressions of miR-744-5p and miR-3140-3p in different cell lines. (C) The expression of miR-138-5p in patient samples was detected by qRT-PCR. (D) FISH assays were conducted for determining the localization of miR-138-5p and LINC00665 in HCT116 and SW480 cells (×1000 magnification). **P < 0.01, n.s.: no significance. [file 12935_2021_2176_MOESM2_ESM.tif]

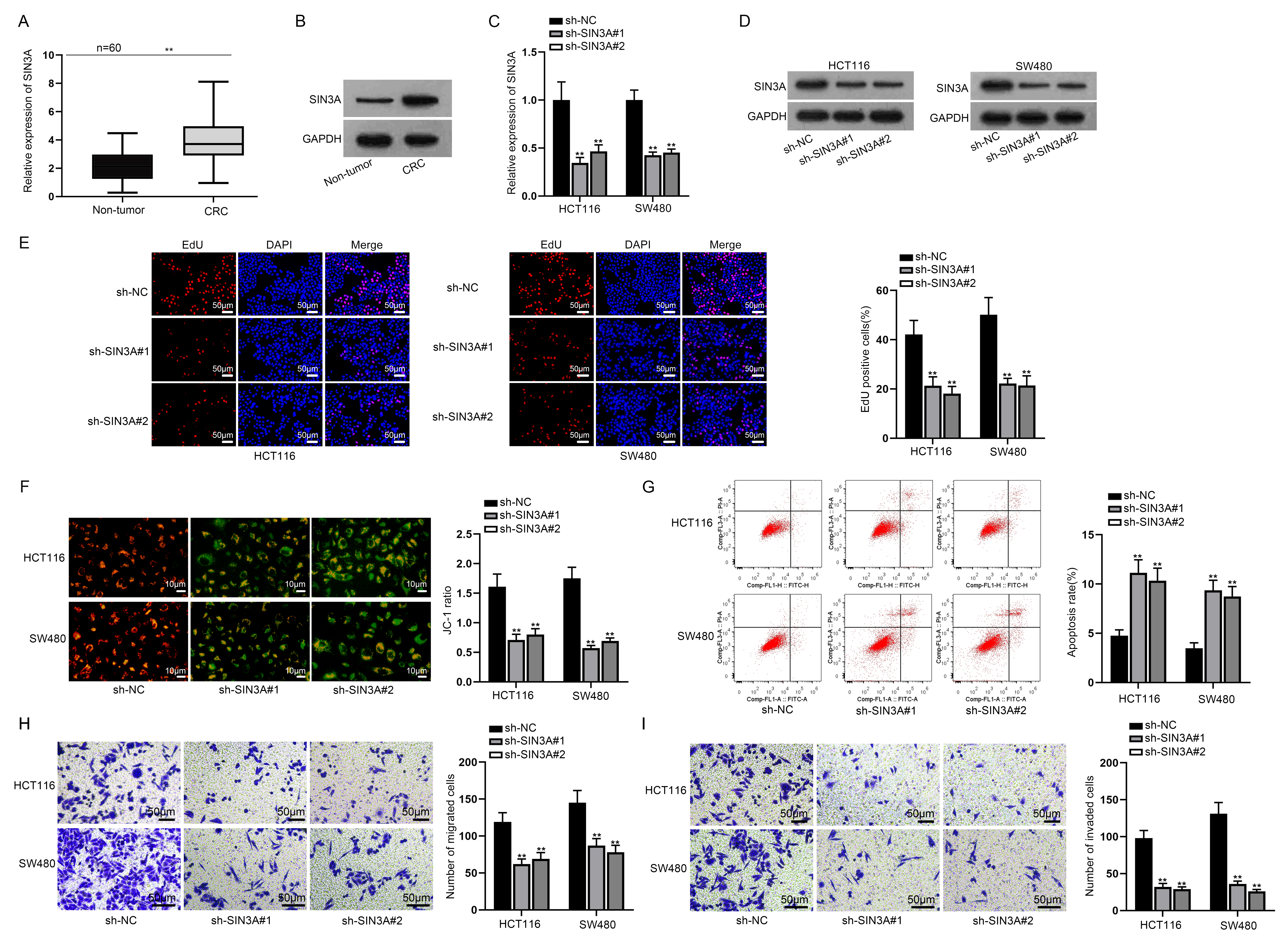

Supplement: Supplementary file 3 — Additional file 3: Figure S3. (A, B) The expression of SIN3A in patient samples was measured by qRT-PCR and WB. (C, D) qRT-PCR and WB were carried out to evaluate the knockdown efficiency of sh-SIN3A. (E) EdU experiments were performed to estimate cell proliferation in response to SIN3A depletion (×100 magnification). (F, G) Cell apoptosis was evaluated by JC-1 (×200 magnification) and flow cytometry experiments in different groups. (H, I) Transwell assays were implemented to detect cell migration and invasion in different groups (×100 magnification). **P < 0.01. [file 12935_2021_2176_MOESM3_ESM.tif]
